# Statistical inconsistency of the unrooted minimize deep coalescence criterion

**DOI:** 10.1371/journal.pone.0251107

**Published:** 2021-05-10

**Authors:** Ayed A. R. Alanzi, James H. Degnan

**Affiliations:** 1 Mathematics Department, College of Science and Human Studies of Hotat Sudair, Majmaah University, Majmaah, Saudi Arabia; 2 Department of Mathematics and Statistics, University of New Mexico, Albuquerque, NM, United States of America; East Carolina University, UNITED STATES

## Abstract

Species trees, which describe the evolutionary relationships between species, are often inferred from gene trees, which describe the ancestral relationships between sequences sampled at different loci from the species of interest. A common approach to inferring species trees from gene trees is motivated by supposing that gene tree variation is due to incomplete lineage sorting, also known as deep coalescence. One of the earliest methods motivated by deep coalescence is to find the species tree that minimizes the number of deep coalescent events needed to explain discrepancies between the species tree and input gene trees. This minimize deep coalescence (MDC) criterion can be applied in both rooted and unrooted settings. where either rooted or unrooted gene trees can be used to infer a rooted species tree. Previous work has shown that MDC is statistically inconsistent in the rooted setting, meaning that under a probabilistic model for deep coalescence, the multispecies coalescent, for some species trees, increasing the number of input gene trees does not make the method more likely to return a correct species tree. Here, we obtain analogous results in the unrooted setting, showing conditions leading to inconsistency of the MDC criterion using the multispecies coalescent model with unrooted gene trees for four taxa and five taxa.

## Introduction

Evolutionary trees estimated at different loci, *gene trees*, vary from one another and from the *species tree*, which represents the history of speciation events. Although there are many causes of such gene tree discordance, one of the most commonly modeled is *deep coalescence*, the failure of two or more gene lineages to coalesce (i.e., be copied from the same gene in the population) in their most recent ancestral population [1, 2]. This phenomenon, also called *incomplete lineage sorting*, is modeled by the *multispecies coalescent*, which makes probabilistic predictions for the probabilities of different gene tree topologies to be observed in a sample of gene trees [3–5]. Although other sources of gene tree heterogeneity are possible, such as gene duplication and loss, hybridization, recombination within genes, and ancient population structure [1, 2, 6, 7] deep coalescence is thought to be quite common for species that underwent rapid radiations [8, 9] and is often used to infer species relationships from gene trees [2, 10, 11].

Species tree inference methods can be based on sequence data or based on analyzing gene trees (e.g., consensus methods). These latter techniques are also called *two-stage methods, meaning that a first stage is estimating the gene trees, and the second stage is combining the information in the gene trees to estimate the species tree. Two-stage techniques are typically computationally faster than sequence-based techniques* and do not have issues with convergence of MCMC algorithms that have arisen for real data sets with many loci for Bayesian methods [12]. Consequently, two-stage methods have remained popular in spite of more sophisticated sequence-based methods. Whether or not a two-stage method is explicitly motivated by the multispecies coalescent, a central concern is whether it is statistically consistent under the multispecies coalescent model. A method is consistent in this setting if the probability that it returns the correct species tree topology tends to 1.0 as the number of loci tends to infinity.

Among two-stage methods for inferring species trees, some have been found to be consistent from known gene trees, while others have been shown to be inconsistent. Two-stage methods that have been shown to be statistically consistent (assuming gene trees are known without error) include rooted triple consensus [13], ASTRAL [14], MP-EST [15], NJ_*st*_ (also called USTAR) [16, 17], and STAR [18]. Some have been found to be statistically inconsistent, including democratic vote [19], greedy consensus [20], matrix representation with parsimony [21], and the minimize deep coalescence (MDC) method in the rooted setting [22]. The MDC method, the focus of this paper, infers the species tree which minimizes the total number of deep coalescence events needed to explain each gene tree, summed over all the input gene trees.

**Fig 1:**
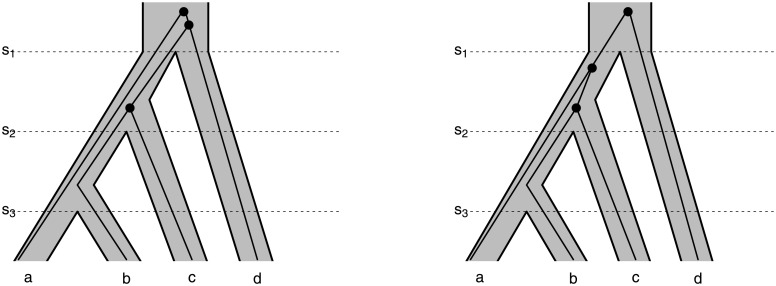
Example species tree—the shaded grey tree with topology (((a,b),c),d)—with two gene trees embedded. In the left example the gene tree is (((B,C),D),A), and on the right the gene tree is (((B,C),A),D). Both gene trees have the same unrooted gene tree, ((B,C),A,D), but different deep coalescence costs. On the left, there are two cases, at time *S*_1_ and *S*_2_ where there are two lineages “exiting” a population (going from the present to the past). In the right-hand example, there is only one population where two lineages “exit”. Thus, the gene tree on the left has deep coalescence cost 2, while the gene tree on the right has deep coalescence cost 1. The unrooted version of MDC minimizes coalescence costs over all possible rootings, so the deep coalescence cost for the unrooted gene tree ((B,C),A,D) is 1 for this species tree.

The idea behind MDC was introduced by Maddison (1997) and was an early method to be implemented for inferring rooted species trees from rooted gene trees motivated by deep coalescence [23–25]. Initially, the method was only applied with rooted gene trees as input, and implementations returned a rooted inferred species tree. To illustrate the idea, consider the species tree and gene trees in Fig 1.

[26], which use approximate Bayesian computation (ABC), is the first method we are aware of to explicitly use the coalescent (i.e., using probabilities from the model) to estimate rooted species trees from unrooted gene trees; however, a version of the minimize deep coalescence method (MDC) was also developed to infer rooted species trees from unrooted gene trees [27]. The idea behind the method is to calculate the MDC score contributed by an unrooted gene tree for a candidate rooted species tree by minimizing the cost over all possible rootings of the gene trees.

Although MDC was one of the first methods to be implemented to infer species trees from gene trees [23], this criterion was found to be statistically inconsistent in the rooted setting (i.e., using rooted gene trees as input) in the same year that its unrooted extension was published [22, 27]. For some species trees, the probability that MDC returns an incorrect species tree tends to 1.0 as the number of input rooted gene trees goes to infinity. Although more accurate methods for inferring species trees have been developed, MDC is still sometimes used to quickly estimate a candidate species tree or phylogenetic network [28], which motivates studying its properties. Currently, there are no fast methods for inferring rooted species trees from unrooted gene trees. Consequently, a possible application of MDC in this setting is to generate candidate trees (particularly because MDC can be used to find sub-optimal trees) to reduce the search time needed for other more computationally intensive methods.

We also note that PhyloNet can use unrooted MDC, which we call UMDC, to return a rooted species tree even in the case of four taxa, although four-taxon gene tree topologies do not identify the rooted species tree under the multispecies coalescent [29]. A theoretical result from Allman et al. (2011) is that the true distribution of unrooted genetic tree topologies can be used to infer rooted species tree when there are five or more taxa, but not when there are only four taxa.

Part of the argument for the identifiability of the rooted species tree from unrooted gene trees is that there are certain inequalities that hold in the gene tree probabilities. For instance, consider distinguishing the two rooted species trees ((((*a*, *b*), *c*), *d*), *e*) versus ((((*a*, *b*), *c*), *e*), *d*). Both species trees have the same unrooted topology but imply different inequalities in some of the unrooted gene tree topology probabilities. For the first species tree, lineage *c* is more likely to coalesce with *d* than *e*; consequently, for the species tree, the unrooted gene tree ((*a*, *b*), *e*, (*c*, *d*)) is more probable than the unrooted gene tree ((*a*, *b*), *d*, (*c*, *e*)). However, for species tree ((((*a*, *b*), *c*), *e*), *d*), these inequalities are reversed. Therefore, observing more unrooted gene trees with topology ((a, b), e, (c, d)) than topology ((*a*, *b*), *d*, (*c*, *e*)) gives evidence favoring the first species tree over the second species tree. Interestingly, for distinguishing the two species trees, the frequency of the matching unrooted gene tree ((*a*, *b*), *c*, (*d*, *e*)) is not helpful. Instead, it is frequencies of nonmatching unrooted gene trees that are useful for inferring the rooted species tree [26]. This paper examines features of the distribution of unrooted topological gene trees that occur under the multispecies coalescent model on a species tree, for deriving asymptotic (i.e., large numbers of loci) UMDC behavior from unrooted gene trees for four taxa and five taxa.

## Results

Let *S* be a binary, rooted species tree on a taxon leaf set *X*, and let λ be a list of branch lengths on *S* measured in coalescent time units. Here, λ_*i*_ = 1 means that branch *i* has a length of *N*_*e*_ generations, where *N*_*e*_ is the effective population size. Let *R*(*X*) be the set of all rooted, binary trees for taxon set *X* and let *U*(*X*) be the set of all unrooted, binary trees for the same taxon set. Let *T* denote a rooted gene tree, and *S*′ a candidate species tree. Let α*(*T*, *S*′) denote the rooted deep coalescence cost (the minimum number of extra lineages) for a rooted gene tree *T* and candidate species tree *S*′. For an unrooted tree *U* with possible rootings *T*^1^, *T*^2^, …, *T*^*k*^, where *k* = 2*n* − 3 and *n* is the number of taxa, the unrooted coalescence cost is

$$\begin{array}{c} \alpha \left(T_{u},S^{\prime }\right)=\underset{i\in \{1,\ldots ,2n-3\}}{\text{min}}\alpha^{*}\left(T^{i},S^{\prime }\right) \end{array}$$

The number of extra lineages at a species boundary is the number of lineages greater than 1 passing from a population to its immediate ancestor. The total number of extra lineages for a gene tree-species tree pair is the sum of extra lineages over the entire species tree (Fig 1).

If G is an observed set of unrooted gene trees, we can think of UMDC as returning the inferred tree $\hat{S}$ that minimizes the average UMDC score:

$$\begin{array}{c} \hat{S}=\underset{S^{\prime }}{\text{arg}\;\text{min}}\;\frac{1}{\left|G\right|}\sum_{U\in G}\alpha \left(U,S^{\prime }\right) \end{array}$$

For a given species tree *S* with branch lengths λ and candidate species tree topology *S*′, we can define the expected UMDC cost as

$$\begin{array}{cc} E[\alpha_{S,\lambda }\left(T,S^{\prime }\right)] & =\sum_{U_{i}\in U\left(X\right)}\alpha \left(U_{i},S^{\prime }\right)P\left(U_{i}|S,\lambda \right)\\ & =\sum_{T\in R(X)}\alpha \left(T,S^{\prime }\right)P\left(T|S,\lambda \right) \end{array}$$

Where we interpret *α*(*T*, *S*′) for a rooted tree *T* as min_*i*_ α*(*T*^*i*^, *S*′), where *T*^*i*^ are the possible re-rootings of *T*. In other words, we interpret *α* applied to a rooted gene tree as minimizing over all possible rootings of the gene tree. The equivalence is due to the fact that we can compute the probability of an unrooted tree by summing over the probabilities of all possible rootings [29].

We note that this expected value depends on the branch lengths of the species tree *S*, but that branch lengths for *S*′ do not need to be specified since only the topology of *S*′ is used (and estimated). If the expected UMDC score is minimized by some *S*′ ≠ *S*, then UMDC is inconsistent since, by the Law of Large Numbers, as the number of loci tends to infinity, the UMDC score will be minimized by a tree other than the species tree with probability 1. To show inconsistency, it is sufficient to find a species trees *S* and *S*′ and branch lengths λ such that *E*[*α*_*S*,λ_(*T*, *S*′)] < *E*[*α*_*S*,λ_(*T*, *S*)].

### Trees with four leaves

Here are three unrooted, binary trees on four leaves. For the species tree, we can consider the two cases of symmetric or asymmetric binary species trees (Table 1). We indicate the *i*^*th*^ *distinctunrootedgenetreetopology*, i = 1, 2, 3, *asT*_*i*_. The asymmetric species tree is also called a caterpillar, denoted *S*_*C*_, and we denote the balanced tree as *S*_*B*_, using one representative labeling for each case. Thus, the species tree is either (*S*_*C*_, λ) = (((*a*, *b*):*x*, *c*):*y*, *d*) or (*S*_*B*_, λ) = ((*a*, *b*):*x*, (*c*, *d*):*y*) where *x* and *y* are branch lengths in coalescent units. The lengths of the external branches are not used.

**Table 1:** UMDC for 4-taxon unrooted gene trees.

Gene tree *T*_*i*_	P(*U*_*i*_|*S*_*C*_, λ)	*α*(*U*_*i*_, *S*_*C*_)	P(*U*_*i*_|*S*_*B*_, λ)	*α*(*U*_*i*_, *S*_*B*_)
*U*_1_ = ((*a*, *b*), *c*, *d*)	$1-\frac{2}{3}e^{-x}$	0	$1-\frac{2}{3}e^{-(x+y)}$	0
*U*_2_ = ((*a*, *c*), *b*, *d*)	$\frac{2}{3}e^{-x}$	1	$\frac{2}{3}e^{-(x+y)}$	2
*U*_3_ = ((*a*, *d*), *b*, *c*)	$\frac{2}{3}e^{-x}$	1	$\frac{2}{3}e^{-(x+y)}$	2

To see how this implies that UMDC is inconsistent, let the true species tree be *S*_*B*_ = (((*a*, *b*):*x*, (*c*, *d*):*y*), then the expected coalescence cost under candidate tree *S*_*C*_ = (((*a*, *b*), *c*), *d*) is (4/3)exp(−(*x* + *y*)). Under candidate tree *S*_*B*_, the expected cost is (8/3)exp(−(*x* + *y*)). Thus, MDC will always give a lower cost to the tree *S*_*C*_ when *S*_*B*_ is the species tree. (Similarly, if *S*_*C*_ is the species tree, UMDC will also give a lower cost to *S*_*C*_ than to *S*_*B*_, regardless of the data.) Thus, MDC is incapable of returning a balanced tree for this scenario. This means that UMDC is inconsistent on four taxa. We also see that if the candidate species tree is *S*′ = (((*a*, *b*), *d*), *c*) (i.e., swapping taxa *c* and *d* in the species tree), then the deep coalescence costs are also 0, 1, and 1 for gene trees *U*_1_, *U*_2_, and *U*_3_, respectively. Thus, regardless of the unrooted gene trees observed, UMDC will give equal scores to *S*_*C*_ and *S*′, so that there is no way to choose one versus the other except for an arbitrary (or random) tie-break.

These results suggest that UMDC should not be used on unrooted four-taxon gene tree topologies. However, this is not unreasonable because it has been shown that under the MSC, four-taxon rooted species trees are not identifiable from unrooted gene trees. Thus, identifying the rooted species tree from four-taxon unrooted gene tree topologies would also not be possible using maximum likelihood, for example. However, rooted species trees are identifiable from unrooted five-taxon gene trees, which we examine next.

### Trees with five leaves

The 15 binary, unrooted trees with five leaves have only one possible shape, whereas rooted trees on five leaves have three shapes, which we call caterpillar, pseudocaterpillar [30], and balanced. We indicate the *i*^*th*^ distinct unrooted gene tree topology, *i* = 1, …, 15, as *T*_*i*_. Although there are 15 possible gene tree topologies, there are 105 rooted species trees possible. An exhaustive approach to UMDC is to compute the UMDC score for all 105 candidate species trees and choose the species tree with the lowest score as the inferred tree.

There are three possible shapes to the rooted species tree when leaf-labels are ignored. The rooted species tree shape is called caterpillar, pseudocaterpillar, or balanced. We use *S*_*C*_, *S*_*P*_ and *S*_*B*_, respectively to denote representative trees from each shape:

$$\begin{array}{cc} S_{C} & =((((a,b):x,c):y,d):z,e)\\ S_{P} & =(((a,b):x,(d,e):y):z,e)\\ S_{B} & =(((a,b):x,c):y),(d,e):z) \end{array}$$

Let *D*_*ijk*_(λ) denote the difference in expected UMDC scores for candidate trees *S*_*j*_ and *S*_*k*_ when the true species tree is *S*_*i*_. Here *i*, *j*, *k* ∈ {*C*, *P*, *B*} to denote caterpillar, pseudocaterpillar, and balanced topologies. Thus,

$$\begin{array}{c} D_{ijk}\left(\lambda \right)=\sum_{h=1}^{15}P\left(U_{h}|S_{i},\lambda \right)\left[\alpha \left(U_{h},S_{j}\right)-\alpha \left(U_{i},S_{k}\right)\right] \end{array}$$

Generally, if *D*_*ijk*_(λ) > 0, then candidate tree *S*_*j*_ has higher expected UMDC score than *S*_*k*_ when the species tree is *S*_*i*_ with branch lengths λ. In particular, if *j* = *i*, then *D*_*iik*_ > 0 means that UMDC will tend to rank the incorrect candidate tree *S*_*k*_ as better than the true tree *S*_*i*_.

The differences in expected values can be obtained from Table 2. To save space, we omit notating the dependence on λ. For example

$$\begin{array}{cc} D_{CBP} & =1\cdot P\left(U_{2}|S_{C}\right)+1\cdot P\left(U_{3}|S_{C}\right)+1\cdot P\left(U_{5}|S_{C}\right)+1\cdot P\left(U_{6}|S_{C}\right)+2\cdot P\left(U_{7}|S_{C}\right)\\ & +2\cdot P\left(U_{8}|S_{C}\right)+\cdots +1\cdot P\left(U_{15}|S_{C}\right). \end{array}$$

**Table 2:** MDC for 5-taxa unrooted gene trees.

Gene tree *U*_*i*_	Pr(*U*_*i*_|*S*_*C*_, λ)	*α*(*U*_*i*_, *S*_*C*_)	Pr(*U*_*i*_|*S*_*P*_, λ)	*α*(*U*_*i*_, *S*_*P*_)	Pr(*U*_*i*_|*S*_*B*_, λ)	*α*(*U*_*i*_, *S*_*B*_)
*U*_1_ = (((a, b), c),(d, e))	$1-\frac{2}{3}\text{X}-\frac{2}{3}\text{Y}+\frac{1}{3}\text{XY}+\frac{1}{18}\text{X}Y^{3}+\frac{1}{90}\text{X}Y^{3}Z^{6}$	0	$1-\frac{2}{3}\text{X}-\frac{2}{3}\text{Y}+\frac{4}{9}\text{XY}-\frac{2}{45}\text{XY}Z^{6}$	0	$1-\frac{2}{3}\text{X}-\frac{2}{3}\text{YZ}+\frac{1}{3}\text{YZ}-\frac{1}{3}\text{XYZ}+\frac{1}{15}\text{X}Y^{3}\text{Z}$	0
*U*_2_ = (((a, b), d),(c, e))	$\frac{1}{3}$Y—$\frac{1}{6}$XY—$\frac{1}{9}$ X*Y*^3^ + $\frac{1}{90}$ X*Y*^3^ *Z*^6^	1	$\frac{1}{3}$Y—$\frac{5}{18}$XY + $\frac{1}{90}$ XY*Z*^6^	1	$\frac{1}{3}$YZ—$\frac{1}{6}$XYZ—$\frac{1}{10}$ X*Y*^3^Z	2
*U*_3_ = (((a, b), e),(c, d))	$\frac{1}{3}$Y—$\frac{1}{6}$XY—$\frac{1}{18}$ X*Y*^3^—$\frac{2}{45}$ X*Y*^3^ *Z*^6^	1	$\frac{1}{3}$Y—$\frac{5}{18}$XY + $\frac{1}{90}$ XY*Z*^6^	1	$\frac{1}{3}$YZ—$\frac{1}{6}$XYZ—$\frac{1}{10}$ X*Y*^3^Z	2
*U*_4_ = (((a, c), b),(d, e))	$\frac{1}{3}$X—$\frac{1}{3}$XY + $\frac{1}{18}$ X*Y*^3^ + $\frac{1}{90}$ X*Y*^3^ *Z*^6^	1	$\frac{1}{3}$X—$\frac{5}{18}$XY + $\frac{1}{90}$ XY*Z*^6^	1	$\frac{1}{3}$X—$\frac{1}{3}$XYZ + $\frac{1}{15}$ X*Y*^3^Z	1
*U*_5_ = (((a, c), d),(b, e))	$\frac{1}{6}$XY—$\frac{1}{9}$ X*Y*^3^ + $\frac{1}{90}$ X*Y*^3^ *Z*^6^	2	$\frac{1}{18}$XY + $\frac{1}{90}$ XY*Z*^6^	2	$\frac{1}{6}$XYZ—$\frac{1}{10}$ X*Y*^3^Z	3
*U*_6_ = (((a, c), e),(b, d))	$\frac{1}{6}$XY—$\frac{1}{18}$ X*Y*^3^—$\frac{2}{45}$ X*Y*^3^ *Z*^6^	2	$\frac{1}{18}$XY + $\frac{1}{90}$ XY*Z*^6^	2	$\frac{1}{6}$XYZ—$\frac{1}{10}$ X*Y*^3^Z	3
*U*_7_ = (((a, d), b),(c, e))	$\frac{1}{18}$ X*Y*^3^ + $\frac{1}{90}$ X*Y*^3^ *Z*^6^	3	$\frac{1}{18}$XY + $\frac{1}{90}$ XY*Z*^6^	2	$\frac{1}{15}$ X*Y*^3^Z	4
*U*_8_ = (((a, d), c),(b, e))	$\frac{1}{18}$ X*Y*^3^ + $\frac{1}{90}$ X*Y*^3^ *Z*^6^	3	$\frac{1}{9}$XY—$\frac{2}{45}$ XY*Z*^6^	2	$\frac{1}{15}$ X*Y*^3^Z	4
*U*_9_ = (((a, d), e),(b, c))	$\frac{1}{6}$XY—$\frac{1}{18}$ X*Y*^3^—$\frac{2}{45}$ X*Y*^3^ *Z*^6^	3	$\frac{1}{18}$XY + $\frac{1}{90}$ XY*Z*^6^	2	$\frac{1}{6}$XYZ—$\frac{1}{10}$ X*Y*^3^Z	3
*U*_10_ = (((a, e), b),(c, d))	$\frac{1}{18}$ X*Y*^3^ + $\frac{1}{90}$ X*Y*^3^ *Z*^6^	3	$\frac{1}{18}$XY + $\frac{1}{90}$ XY*Z*^6^	2	$\frac{1}{15}$ X*Y*^3^Z	4
*U*_11_ = (((a, e), c),(b, d))	$\frac{1}{18}$ X*Y*^3^ + $\frac{1}{90}$ X*Y*^3^ *Z*^6^	3	$\frac{1}{9}$XY—$\frac{2}{45}$ XY*Z*^6^	2	$\frac{1}{15}$ X*Y*^3^Z	4
*U*_12_ = (((a, e), d),(b, c))	$\frac{1}{6}$XY—$\frac{1}{9}$ X*Y*^3^ + $\frac{1}{90}$ X*Y*^3^ *Z*^6^	2	$\frac{1}{18}$XY + $\frac{1}{90}$ XY*Z*^6^	2	$\frac{1}{6}$XYZ—$\frac{1}{10}$ X*Y*^3^Z	3
*U*_13_ = (((b, c), a),(d, e))	$\frac{1}{3}$X—$\frac{1}{3}$XY + $\frac{1}{18}$ X*Y*^3^ + $\frac{1}{90}$ X*Y*^3^ *Z*^6^	1	$\frac{1}{3}$X—$\frac{5}{18}$XY + $\frac{1}{90}$ XY*Z*^6^	1	$\frac{1}{3}$X—$\frac{1}{3}$XYZ + $\frac{1}{15}$ X*Y*^3^Z	2
*U*_14_ = (((b, d), a),(c, e))	$\frac{1}{18}$ X*Y*^3^ + $\frac{1}{90}$ X*Y*^3^ *Z*^6^	3	$\frac{1}{18}$XY + $\frac{1}{90}$ XY*Z*^6^	2	$\frac{1}{15}$ X*Y*^3^Z	4
*U*_15_ = (((b, e), a),(c, d))	$\frac{1}{18}$ X*Y*^3^ + $\frac{1}{90}$ X*Y*^3^ *Z*^6^	3	$\frac{1}{18}$XY + $\frac{1}{90}$ XY*Z*^6^	2	$\frac{1}{15}$ X*Y*^3^Z	4

We note that the coefficients of *P*(*U*_*i*_|*S*_*C*_) in *D*_*CBP*_ are all positive, indicating that the balanced tree has a higher cost than the pseudocaterpillar when the species tree is *S*_*C*_. This holds regardless of the choice of branch lengths λ. Similarly, we see that for any branch lengths λ,

$$\begin{array}{c} D_{CBP}>0,\;D_{CCP}>0,\;D_{BBP}>0,\;D_{BCB}>0,\;D_{PBC}>0, \end{array}$$

$$\begin{array}{c} D_{PPB}<0,\;D_{PPC}<0 \end{array}$$

The theoretical expected values show that, for example, if the species tree is a caterpillar, then at least one pseudocaterpillar has lower expected deep coalescence cost than the matching caterpillar species tree, and consequently UMDC is not consistent for recovering the true species tree. Similarly, if the species tree is balanced, then at least one pseudocaterpillar tree always has lower expected coalescence cost the true species tree. In both cases, UMDC will be misleading, tending to return an incorrect species tree as more loci are examined. We note that these relationships hold regardless of the branch lengths of the species tree. Remarkably, these inequalities hold not only asymptotically as the UMDC score approaches its expected value, but even for finite numbers of loci (but using non-strict inequalities). For example, let the number of 5-taxon trees in a sample be (*n*_1_, *n*_2_, …, *n*_15_) where the subscript indexes the unrooted topologies from Table 2. If the species tree is *S*_*C*_, then the UMDC score for $S_{C}^{\prime }$ minus that for $S_{P}^{\prime }$ is

$$\begin{array}{c} S_{C}^{\prime }-S_{P}^{\prime }=n_{7}+n_{8}+n_{9}+n_{10}+n_{11}+n_{14}+n_{15}\geq 0 \end{array}$$

Since this is always greater than or equal to 0, the matching tree can never have better deep coalescence cost than $S_{P}^{\prime }$ (if none of these 7 topologies are observed, the UMDC scores will be tied for these two candidate species trees). If the species tree is *S*_*B*_, then the situation is even worse, with

$$\begin{array}{c} S_{B}^{\prime }-S_{P}^{\prime }=n_{2}+n_{3}+n_{5}+n_{6}+2n_{7}+2n_{8}+n_{9}+2n_{10}+2n_{11}+n_{12}+n_{13}+2n_{14}+2n_{15}\geq 0. \end{array}$$

This shows that MDC is misleading for five taxa if the species tree does not have pseudocaterpillar topology.

These examples are sufficient to show that UMDC is inconsistent for trees with five taxa. However, to better understand the behavior of UMDC, it is helpful to also understand the MDC costs for other true species tree and candidate species tree combinations (S1 and S2 Tables in S1 Appendix). An interesting question here is whether UMDC will tend to perform well within a particular unlabeled shape. For example, if it is known (or believed) that the species tree has a particular shape (for example, a caterpillar), will UMDC pick the correct species tree if it restricted to the correct unlabeled shape? This situation can arise in particular if alternative rootings lead to two candidate trees that have the same shape. In this case, we can examine in which cases UMDC might or might not be misleading. A potential use here is that UMDC is used to generate candidate species trees; it could return the best species tree for each tree shape, and more computationally intensive methods could then use these as starting trees.

From S1 and S2 Tables in S1 Appendix, we see that UMDC cannot distinguish two caterpillar trees with the outgroup swapped with the taxon that is an outgroup to all other ingroup taxa, for example species trees (((*a*, *b*), *c*), *d*), *e*) and ((((*a*, *b*), *c*), *e*), *d*) will have exactly the same UMDC score for any data set. To check if, for example, these are expected to be the best scoring caterpillar trees when the species tree is *S*_*C*_, we can use expected values. For example, to compare the expected score for $S_{1}^{\prime }=\left(\left(\left(\left(a,b\right),c\right),d\right),e\right)$ with $S_{3}^{\prime }=\left(\left(\left(\left(a,b\right),d\right),c\right),e\right)$, we note that the MDC cost is the same for these candidate species trees for unrooted gene trees *U*_3_, *U*_6_, *U*_9_, *U*_10_, and *U*_15_. The difference in expected values therefore depends on the other 10 trees. Collecting terms, the difference is

$$\begin{array}{cc} f(X,Y,Z) & =E[\alpha \left(U,S_{3}^{\prime }\right)-\alpha \left(U,S_{1}^{\prime }\right)]\\ & =-1-X(2/3-2/3+2/3)+Y(2/3+1/3)+XY(-1/3-1/6+\cdots +2/3)\\ & \;\;+XY^{3}\left(1/18+\cdots +2/18\right)+XY^{3}Z^{6}\left(1/90+\cdots +2/90\right)\\ & =-1-\left(2/3\right)X+Y+XY/6+4XY^{3}/18-5XY^{3}Z^{6}/90 \end{array}$$

Because *f*(*X*, *Y*, *Z*) is decreasing in Z, and therefore *f*(*X*, *Y*, *Z*) < *f*(*X*, *Y*, 0) for all *Z*, a sufficient condition to show that that *S*_*C*_ has lower expected score than $S_{3}^{\prime }$ is that

$$\begin{array}{c} -1-\left(2/3\right)X+Y+XY/6+4XY^{3}/18<0 \end{array}$$

Note that the expression is equivalent to

$$\begin{array}{c} -1-X(2/3+Y/6+4Y^{3}/18)+Y \end{array}$$

Because −1 + *Y* < 0 and the term in parenthesis is positive, this shows that the difference in expected UMDC costs is negative; hence *S*_*C*_ is expected to be preferred over $S_{3}^{\prime }$ when *S*_*C*_ is the species tree.

Similar arguments can be made, although tediously, to show that *S*_*C*_ has the lowest expected UMDC score (although tied with $S_{2}^{\prime }$) among all caterpillar candidate species trees. Because of the ties in the UMDC costs for pairs of candidate caterpillar species trees, this requires evaluating 29 such inequalities.

### Simulation

To illustrate the theoretical results, we simulated gene trees from the species trees *S*_*C*_, *S*_*P*_, and *S*_*B*_ using hybrid-Lambda [31], where all internal branch lengths were 1.0 coalescent units, which allows a moderate amount of incomplete lineage sorting. The species trees were

$$\begin{array}{cc} S_{C} & =((((a:1.0,b:1.0):1.0,c:2.0):1.0,d:3.0):1.0,e:4.0)\\ S_{P} & =(((a:1.0,b:1.0):1.0,(c:1.0,d:1.0):1.0):1.0,e:4.0)\\ S_{B} & =(((a:1.0,b:1.0):1.0,c:2.0):1.0,(d:2.0,e:2.0):1.0) \end{array}$$

These species trees were also repeated with each branch length multiplied by 0.1 to investigate the effect of shorter branch lengths. For each species tree, independent simulations were run with 50, 100, 200, 400, and 800 loci. For each combination of species tree and number of loci, a set of gene trees was simulated using hybrid-Lambda [31]. Species trees were estimated directly from these known gene trees, and a second set of simulations was done using estimated gene trees. To estimate gene trees, DNA sequences were simulated from the gene trees using seq-gen version 1.3.2x [32] with 500 nucleotides per locus and base frequencies of 0.3, 0.2, 0.2, and 0.3 for nucleotides A, C, G, and T, respectively with a mutation rate of *θ* = 0.01 under a *GTR* + Γ + *I* model with four variable rates and 10% invariable sites. Gene trees were then estimated as unrooted using phyml version 20120412 [33] under the correct model. Each of these settings was repeated 100 times, and the proportion of times various tree topologies were inferred was recorded. The UMDC tree was obtained from phylonet using the command Infer_ST_MDC_UR. In case of a tie between highest scoring species trees, a tree was picked uniformly at random as the species tree estimate.

**Fig 2:**
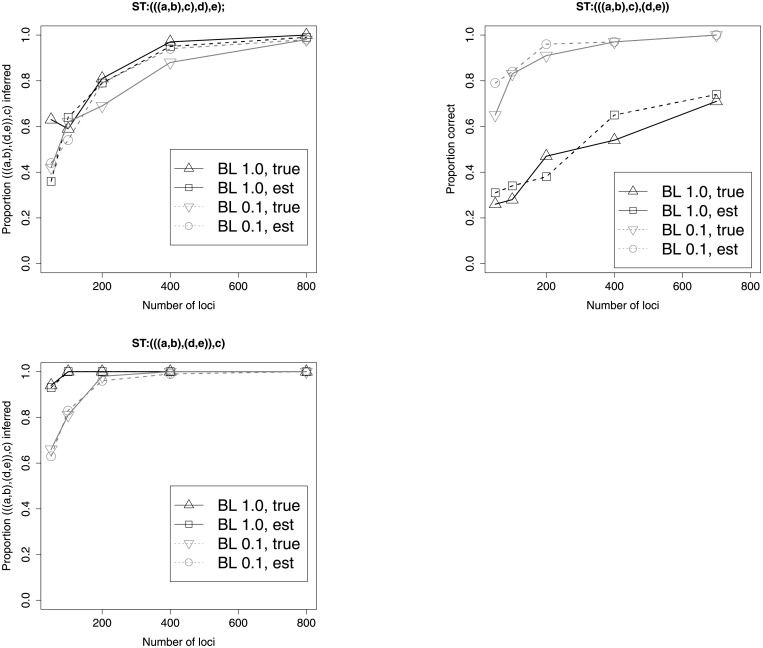
Simulation for species trees *S*_*C*_, *S*_*B*_, and *S*_*P*_. The proportion indicates the number of times out of 100 that the species tree topology (((*a*, *b*), *c*), (*d*, *e*)) was inferred. “BL 1.0” means that internal branches, as well as pendant branches leading to taxa *a* and *b* had length 1.0, while remaining pendant edges had lengths needed to make the trees ultrametric. “BL 0.1” had all branch lengths multiplied by 0.1 in the species tree. The terms “true” and “est” refer to whether gene trees were estimated from simulated DNA sequences or the actual simulated gene trees were used.

As predicted by the theory, regardless of the species tree, the pseudocaterpillar shape tends to be the tree inferred, with probability approaching 1.0 as the sample size (number of loci) increases (Fig 2). In cases where a non-pseudocaterpillar was inferred, this was due to randomly picking one of the trees tied for best UMDC score. Ties for the best MDC score were less likely with larger sample sizes, with 70% of cases (out of 500) with 50 loci having three trees tied for best when the species tree was *S*_*C*_, and 1.2% of cases being tied with 800 loci for *S*_*C*_. Results were similar for *S*_*B*_, but there were much fewer ties for best tree for the pseudocaterpillar species tree, *S*_*P*_. For *S*_*P*_, 7% of cases had a tree tied for best with 50 loci, and the best tree was always unique with 100 or more loci.

We investigated the effects of using shorter branch lengths (multiplying each branch length by 0.1) and of estimating gene trees from DNA sequences to investigate the effects of both greater gene tree heterogeneity due to shorter branches and gene tree estimation error. For the caterpillar species tree, there was very little effect of either gene tree estimation error or shortening species tree branches (Fig 2). For all species trees, estimation error had little effect on the inference. When branches were multiplied by 0.1, convergence to the incorrect species tree was more rapid for the balanced tree, while convergence to the correct tree was slightly slower when the species tree was a pseudocaterpillar.

## Discussion

In this paper, we used a method of considering expected values of scores to show properties of the unrooted version of MDC. This approach of using expected values has also been used to show inconsistency of the original MDC criterion [22] and matrix representation with parsimony [21]. Although we only showed inconsistency for four- and five-taxon trees, the results apply straightforwardly to larger trees. For example, “caterpillarization” is a technique of making some branches long enough while keeping others short that the distribution of gene trees resembles the the distribution found from a caterpillar species tree, and all the results would apply to these larger trees as well lemmas 3 and 5 in [34]. This means that for larger species trees, there exist branch lengths for which UMDC will be misleading.

To explain this idea in more detail, suppose a six-taxon species tree has topology ((((*a*, *b*), *c*), *d*), (*e*, *f*)). If the branch leading from the most recent common ancestor (MRCA) of *e* and *f* to the root is long, then lineages sampled from *e* and *f* will almost certainly coalesce more recently than the root of the tree. Consequently, almost all gene trees from this species tree will have (*e*, *f*) as a cluster, and the species tree will have very similar properties as a species tree ((((*a*, *b*), *c*), *d*), *x*) with *x* replaced by (*e*, *f*). Consequently, if this branch is sufficiently long, unrooted gene tree distributions on taxa *a*–*f* are concentrated on just the 15 unrooted topologies that occur on five-taxon trees, and we can predict what species tree inference methods will do based on their behavior on 5-taxon trees. Another example is the 6-taxon pseudocaterpillar species tree (((*a*, *b*), (*c*, *d*), *e*), *f*). This tree can be caterpillarized by letting the branch leading from the MRCA of *c* and *d* to the MRCA of *a*, *b*, *c*, and *d* be sufficiently long. Replacing (*c*, *d*) with *x* in this tree, it resembles a five-taxon caterpillar ((((*a*, *b*), *x*), *e*), *f*) for which we can expect UMDC to prefer tree (((*a*, *b*), (*e*, *f*)), *x*) = ((*a*, *b*), ((*c*, *d*), (*e*, *f*)). This approach is sufficient to show that any tree that can be caterpillarized to a five-taxon caterpillar tree can be misleading in the ways shown in this paper given certain branch lengths. A more detailed proof for this particular six-taxon tree would show that of the 105 6-taxon topologies, for any given *ϵ* > 0, branch lengths in the species tree can be chosen such that the probability is greater than 1 − *ϵ* that the probability that the gene tree is one of the 15 trees concentrated on taxa *a*, *b*, *x*, *e*, and *f*.

Similarly, many species trees with more than five taxa can mimic the behavior of the 5-taxon balanced tree given certain branch lengths. For example, the species tree (((*a*, *b*), *c*), (*d*, (*e*, *f*))) will mimic the 5-taxon balanced tree in this paper if the branch leading from the MRCA of *e* and *f* to the MRCA of *d*, *e*, and *f* is sufficiently long. A general proof of inconsistency for UMDC for trees with 6–8 taxa would examine some special cases like these and show that there are branch lengths which could make a tree mimic the 5-taxon caterpillar or balanced trees. Trees with 9 or more taxa can always be caterpillarized to a 5-taxon caterpillar [21, 34]

Although the results are negative, MDC and UMDC can still be used to quickly generate starting trees when searching for species trees using other methods. An advantage of MDC and UMDC here is that they can rank trees by score, and therefore return suboptimal trees. Since MDC has a shape bias, tending to make it return more balanced trees [35], it is not surprising that there is a shape bias for UMDC as well. The bias for UMDC is surprisingly extreme however in that UMDC always prefers certain shapes (the pseudocaterpillar for five taxa) for any data. The results of this paper suggest that due to the shape bias of UMDC, a preferable method to generating starting trees might be to return the set of optimal trees within each unlabeled shape. The impact that this could have on species tree inference we leave to future work.

## Supporting information

S1 Appendix (PDF)
