# Correction: Statistical inconsistency of the unrooted minimize deep coalescence criterion

**DOI:** 10.1371/journal.pone.0256189

**Published:** 2021-08-09

**Authors:** Ayed R. A. Alanzi, James H. Degnan

The first author’s name appears incorrectly. The correct name is: Ayed R. A. Alanzi. The correct citation is: Alanzi ARA, Degnan JH (2021) Statistical inconsistency of the unrooted minimize deep coalescence criterion. PLoS ONE 16(5): e0251107. https://doi.org/10.1371/journal.pone.0251107

There is an error in affiliation 1 for author Ayed R. A. Alanzi. The correct affiliation 1 is: Mathematics Department, College of Science and Human Studies of Hotat Sudair, Majmaah University, Al-Majmaah, Saudi Arabia.
