# Supplementary material for: Statistical inconsistency of the unrooted minimize deep coalescence criterion
Source: PLoS One. 2021 May 10;16(5):e0251107. doi: 10.1371/journal.pone.0251107 (PMC8109837; doi:10.1371/journal.pone.0251107)
Supplement: S1 Appendix — (PDF) [file pone.0251107.s001.pdf]

S1 Table. Table of costs. The (i, j)th entry is $\alpha(U_j, S_i')$.

((((a,b),c),d),e);	0	1	1	1	2	2	3	3	2	3	3	2	1	3	3
((((a,b),c),e),d);	0	1	1	1	2	2	3	3	2	3	3	2	1	3	3
((((a,b),d),c),e);	1	0	1	3	3	2	1	2	2	3	2	3	3	1	3
((((a,b),d),e),c);	1	0	1	3	3	2	1	2	2	3	2	3	3	1	3
((((a,b),e),c),d);	1	1	0	3	2	3	3	2	3	1	2	2	3	3	1
((((a,b),e),d),c);	1	1	0	3	2	3	3	2	3	1	2	2	3	3	1
((((a,c),b),d),e);	1	2	2	0	1	1	3	3	2	3	3	2	1	3	3
((((a,c),b),e),d);	1	2	2	0	1	1	3	3	2	3	3	2	1	3	3
((((a,c),d),b),e);	3	3	2	1	0	1	2	1	2	2	3	3	3	3	1
((((a,c),d),e),b);	3	3	2	1	0	1	2	1	2	2	3	3	3	3	1
((((a,c),e),b),d);	3	2	3	1	1	0	2	3	3	2	1	2	3	1	3
((((a,c),e),d),b);	3	2	3	1	1	0	2	3	3	2	1	2	3	1	3
((((a,d),b),c),e);	2	1	2	3	3	2	0	1	1	3	2	3	3	1	3
((((a,d),b),e),c);	2	1	2	3	3	2	0	1	1	3	2	3	3	1	3
((((a,d),c),b),e);	3	3	2	2	1	2	1	0	1	2	3	3	3	3	1
((((a,d),c),e),b);	3	3	2	2	1	2	1	0	1	2	3	3	3	3	1
((((a,d),e),b),c);	2	3	3	2	3	3	1	1	0	2	2	1	1	3	3
((((a,d),e),c),b);	2	3	3	2	3	3	1	1	0	2	2	1	1	3	3
((((a,e),b),c),d);	2	2	1	3	2	3	3	2	3	0	1	1	3	3	1
((((a,e),b),d),c);	2	2	1	3	2	3	3	2	3	0	1	1	3	3	1
((((a,e),c),b),d);	3	2	3	2	2	1	2	3	3	1	0	1	3	1	3
((((a,e),c),d),b);	3	2	3	2	2	1	2	3	3	1	0	1	3	1	3
((((a,e),d),b),c);	2	3	3	2	3	3	2	2	1	1	1	0	1	3	3
((((a,e),d),c),b);	2	3	3	2	3	3	2	2	1	1	1	0	1	3	3
((((b,c),a),d),e);	1	2	2	1	2	2	3	3	1	3	3	1	0	3	3
((((b,c),a),e),d);	1	2	2	1	2	2	3	3	1	3	3	1	0	3	3
((((b,c),d),a),e);	3	3	2	3	3	2	3	3	1	1	1	0	1	2	2
((((b,c),d),e),a);	3	3	2	3	3	2	3	3	1	1	1	0	1	2	2
((((b,c),e),a),d);	3	2	3	3	2	3	1	1	0	3	3	1	1	2	2
((((b,c),e),d),a);	3	2	3	3	2	3	1	1	0	3	3	1	1	2	2
((((b,d),a),c),e);	2	1	2	3	3	1	1	2	2	3	1	3	3	0	3
((((b,d),a),e),c);	2	1	2	3	3	1	1	2	2	3	1	3	3	0	3
((((b,d),c),a),e);	3	3	2	3	3	1	3	3	2	1	0	1	2	1	2
((((b,d),c),e),a);	3	3	2	3	3	1	3	3	2	1	0	1	2	1	2
((((b,d),e),a),c);	2	3	3	1	1	0	3	2	3	3	1	3	2	1	2
((((b,d),e),c),a);	2	3	3	1	1	0	3	2	3	3	1	3	2	1	2
((((b,e),a),c),d);	2	2	1	3	1	3	3	1	3	1	2	2	3	3	0
((((b,e),a),d),c);	2	2	1	3	1	3	3	1	3	1	2	2	3	3	0
((((b,e),c),a),d);	3	2	3	3	1	3	1	0	1	3	3	2	2	2	1
((((b,e),c),d),a);	3	2	3	3	1	3	1	0	1	3	3	2	2	2	1
((((b,e),d),a),c);	2	3	3	1	0	1	3	1	3	3	2	3	2	2	1
((((b,e),d),c),a);	2	3	3	1	0	1	3	1	3	3	2	3	2	2	1
((((c,d),a),b),e);	3	3	1	2	1	2	2	1	2	1	3	3	3	3	0
((((c,d),a),e),b);	3	3	1	2	1	2	2	1	2	1	3	3	3	3	0
((((c,d),b),a),e);	3	3	1	3	3	2	3	3	2	0	1	1	2	2	1
((((c,d),b),e),a);	3	3	1	3	3	2	3	3	2	0	1	1	2	2	1
((((c,d),e),a),b);	1	1	0	2	3	3	2	3	3	1	3	3	2	2	1
((((c,d),e),b),a);	1	1	0	2	3	3	2	3	3	1	3	3	2	2	1
((((c,e),a),b),d);	3	1	3	2	2	1	1	3	3	2	1	2	3	0	3
((((c,e),a),d),b);	3	1	3	2	2	1	1	3	3	2	1	2	3	0	3
((((c,e),b),a),d);	3	1	3	3	2	3	0	1	1	3	3	2	2	1	2
((((c,e),b),d),a);	3	1	3	3	2	3	0	1	1	3	3	2	2	1	2
((((c,e),d),a),b);	1	0	1	2	3	3	1	3	3	2	3	3	2	1	2
((((c,e),d),b),a);	1	0	1	2	3	3	1	3	3	2	3	3	2	1	2

S2 Table. Table of costs. The (i, j)th entry is $\alpha(U_j, S_i')$.

((((d,e),a),b),c);	1	3	3	1	3	3	2	2	1	2	2	1	0	3	3
((((d,e),a),c),b);	1	3	3	1	3	3	2	2	1	2	2	1	0	3	3
((((d,e),b),a),c);	1	3	3	0	1	1	3	2	3	3	2	3	1	2	2
((((d,e),b),c),a);	1	3	3	0	1	1	3	2	3	3	2	3	1	2	2
((((d,e),c),a),b);	0	1	1	1	3	3	2	3	3	2	3	3	1	2	2
((((d,e),c),b),a);	0	1	1	1	3	3	2	3	3	2	3	3	1	2	2
(((a,b),(c,d)),e);	1	1	0	2	2	2	2	2	2	1	2	2	2	2	1
(((a,c),(b,d)),e);	2	2	2	1	1	0	2	2	2	2	1	2	2	1	2
(((a,d),(b,c)),e);	2	2	2	2	2	2	1	1	0	2	2	1	1	2	2
(((a,b),(c,e)),d);	1	0	1	2	2	2	1	2	2	2	2	2	2	1	2
(((a,c),(b,e)),d);	2	2	2	1	0	1	2	1	2	2	2	2	2	2	1
(((a,e),(b,c)),d);	2	2	2	2	2	2	2	2	1	1	1	0	1	2	2
(((a,b),(d,e)),c);	0	1	1	1	2	2	2	2	2	2	2	2	1	2	2
(((a,d),(b,e)),c);	2	2	2	2	1	2	1	0	1	2	2	2	2	2	1
(((a,e),(b,d)),c);	2	2	2	2	2	1	2	2	2	1	0	1	2	1	2
(((a,c),(d,e)),b);	1	2	2	0	1	1	2	2	2	2	2	2	1	2	2
(((a,d),(c,e)),b);	2	1	2	2	2	2	0	1	1	2	2	2	2	1	2
(((a,e),(c,d)),b);	2	2	1	2	2	2	2	2	2	0	1	1	2	2	1
(((b,c),(d,e)),a);	1	2	2	1	2	2	2	2	1	2	2	1	0	2	2
(((b,d),(c,e)),a);	2	1	2	2	2	1	1	2	2	2	1	2	2	0	2
(((b,e),(c,d)),a);	2	2	1	2	1	2	2	1	2	1	2	2	2	2	0
(((a,b),c),(d,e));	0	2	2	1	3	3	4	4	3	4	4	3	1	4	4
(((a,c),b),(d,e));	1	3	3	0	2	2	4	4	3	4	4	3	1	4	4
(((b,c),a),(d,e));	1	3	3	1	3	3	4	4	2	4	4	2	0	4	4
(((a,b),d),(c,e));	2	0	2	4	4	3	1	3	3	4	3	4	4	1	4
(((a,d),b),(c,e));	3	1	3	4	4	3	0	2	2	4	3	4	4	1	4
(((b,d),a),(c,e));	3	1	3	4	4	2	1	3	3	4	2	4	4	0	4
(((a,c),d),(b,e));	4	4	3	2	0	2	3	1	3	3	4	4	4	4	1
(((a,d),c),(b,e));	4	4	3	3	1	3	2	0	2	3	4	4	4	4	1
(((c,d),a),(b,e));	4	4	2	3	1	3	3	1	3	2	4	4	4	4	0
(((b,c),d),(a,e));	4	4	3	4	4	3	4	4	2	1	1	0	2	3	3
(((b,d),c),(a,e));	4	4	3	4	4	2	4	4	3	1	0	1	3	2	3
(((c,d),b),(a,e));	4	4	2	4	4	3	4	4	3	0	1	1	3	3	2
(((a,b),e),(c,d));	2	2	0	4	3	4	4	3	4	1	3	3	4	4	1
(((a,e),b),(c,d));	3	3	1	4	3	4	4	3	4	0	2	2	4	4	1
(((b,e),a),(c,d));	3	3	1	4	2	4	4	2	4	1	3	3	4	4	0
(((a,c),e),(b,d));	4	3	4	2	2	0	3	4	4	3	1	3	4	1	4
(((a,e),c),(b,d));	4	3	4	3	3	1	3	4	4	2	0	2	4	1	4
(((c,e),a),(b,d));	4	2	4	3	3	1	2	4	4	3	1	3	4	0	4
(((b,c),e),(a,d));	4	3	4	4	3	4	1	1	0	4	4	2	2	3	3
(((b,e),c),(a,d));	4	3	4	4	2	4	1	0	1	4	4	3	3	3	2
(((c,e),b),(a,d));	4	2	4	4	3	4	0	1	1	4	4	3	3	2	3
(((a,d),e),(b,c));	3	4	4	3	4	4	2	2	0	3	3	1	1	4	4
(((a,e),d),(b,c));	3	4	4	3	4	4	3	3	1	2	2	0	1	4	4
(((d,e),a),(b,c));	2	4	4	2	4	4	3	3	1	3	3	1	0	4	4
(((b,d),e),(a,c));	3	4	4	1	1	0	4	3	4	4	2	4	3	2	3
(((b,e),d),(a,c));	3	4	4	1	0	1	4	2	4	4	3	4	3	3	2
(((d,e),b),(a,c));	2	4	4	0	1	1	4	3	4	4	3	4	2	3	3
(((c,d),e),(a,b));	1	1	0	3	4	4	3	4	4	2	4	4	3	3	2
(((c,e),d),(a,b));	1	0	1	3	4	4	2	4	4	3	4	4	3	2	3
(((d,e),c),(a,b));	0	1	1	2	4	4	3	4	4	3	4	4	2	3	3

To quickly compute differences in expected value for the caterpillar species tree, let A_C. be 6×15 matrices of coefficients of the terms $\mathbf{z} = (1, X, Y, XY, XY^3, XY^3Z^6)$, where entry (i, j) is the coefficient of z_i for gene tree U_j. Here the coefficient matrices can be taken from Table 2. For example A_C is the matrix

$$A_C = \begin{bmatrix} 1 & 0 & 0 & 0 & 0 & 0 & 0 & 0 & 0 & 0 & 0 & 0 & 0 & 0 & 0 \\ -\frac{2}{3} & 0 & 0 & \frac{1}{3} & 0 & 0 & 0 & 0 & 0 & 0 & 0 & 0 & \frac{1}{3} & 0 & 0 \\ -\frac{2}{3} & \frac{1}{3} & \frac{1}{3} & 0 & 0 & 0 & 0 & 0 & 0 & 0 & 0 & 0 & 0 & 0 & 0 \\ \frac{1}{3} & -\frac{1}{6} & -\frac{1}{6} & -\frac{1}{3} & \frac{1}{6} & \frac{1}{6} & 0 & 0 & \frac{1}{6} & 0 & 0 & \frac{1}{6} & -\frac{1}{3} & 0 & 0 \\ \frac{1}{18} & -\frac{2}{18} & -\frac{1}{18} & \frac{1}{18} & -\frac{2}{18} & -\frac{1}{18} & \frac{1}{18} & \frac{1}{18} & -\frac{1}{18} & \frac{1}{18} & \frac{1}{18} & -\frac{2}{18} & \frac{1}{18} & \frac{1}{18} & \frac{1}{18} \\ \frac{1}{90} & \frac{1}{90} & -\frac{4}{90} & \frac{1}{90} & \frac{1}{90} & -\frac{4}{90} & \frac{1}{90} & \frac{1}{90} & -\frac{4}{90} & \frac{1}{90} & \frac{1}{90} & \frac{1}{90} & \frac{1}{90} & \frac{1}{90} & \frac{1}{90} \end{bmatrix}$$

For the pseudocaterpillar species tree, S_P, we define A_P similarly to A_C, except that it is 5×15 since there are only 5 terms needed: 1, X, Y, XY, and XYZ^6.

$$A_P = \begin{bmatrix} 1 & 0 & 0 & 0 & 0 & 0 & 0 & 0 & 0 & 0 & 0 & 0 & 0 & 0 & 0 \\ -\frac{2}{3} & 0 & 0 & \frac{1}{3} & 0 & 0 & 0 & 0 & 0 & 0 & 0 & 0 & \frac{1}{3} & 0 & 0 \\ -\frac{2}{3} & \frac{1}{3} & \frac{1}{3} & 0 & 0 & 0 & 0 & 0 & 0 & 0 & 0 & 0 & 0 & 0 & 0 \\ \frac{4}{9} & -\frac{5}{18} & -\frac{5}{18} & -\frac{5}{18} & \frac{1}{18} & \frac{1}{18} & \frac{1}{18} & \frac{1}{9} & \frac{1}{18} & \frac{1}{18} & \frac{1}{9} & \frac{1}{18} & -\frac{5}{18} & \frac{1}{18} & \frac{1}{18} \\ -\frac{4}{90} & \frac{1}{90} & \frac{1}{90} & \frac{1}{90} & \frac{1}{90} & \frac{1}{90} & \frac{1}{90} & -\frac{4}{90} & \frac{1}{90} & \frac{1}{90} & -\frac{4}{90} & \frac{1}{90} & \frac{1}{90} & \frac{1}{90} & \frac{1}{90} \end{bmatrix}$$

The coefficient matrix for the balanced tree is also 5×15, with rows 1 through 5 corresponding to the terms $1, X, YZ, XYZ$, and XY^3Z

$$A_B = \begin{bmatrix} 1 & 0 & 0 & 0 & 0 & 0 & 0 & 0 & 0 & 0 & 0 & 0 & 0 & 0 & 0 \\ -\frac{2}{3} & 0 & 0 & \frac{1}{3} & 0 & 0 & 0 & 0 & 0 & 0 & 0 & 0 & \frac{1}{3} & 0 & 0 \\ -\frac{2}{3} & \frac{1}{3} & \frac{1}{3} & 0 & 0 & 0 & 0 & 0 & 0 & 0 & 0 & 0 & 0 & 0 & 0 \\ \frac{1}{3} & -\frac{1}{6} & -\frac{1}{6} & -\frac{1}{3} & \frac{1}{6} & \frac{1}{6} & 0 & 0 & \frac{1}{6} & 0 & 0 & \frac{1}{6} & -\frac{1}{3} & 0 & 0 \\ \frac{1}{15} & -\frac{1}{10} & -\frac{1}{10} & \frac{1}{15} & -\frac{1}{10} & -\frac{1}{10} & \frac{1}{15} & \frac{1}{15} & -\frac{1}{10} & \frac{1}{15} & \frac{1}{15} & -\frac{1}{10} & \frac{1}{15} & \frac{1}{15} & \frac{1}{15} \end{bmatrix}$$

To determine the expected difference in MDC scores for two trees, let $\mathbf{B} = (b_{ij})$ be the 105×15 matrix obtained from S1 and S2 Tables, where b_{ij} is the MDC cost for gene tree U_j under candidate species tree T_i. Let $\mathbf{b}_i$ be the ith row of $\mathbf{B}$, treated as a column vector. Then the expected difference in MDC costs comparing tree T_i and T_j is

$$E[\alpha(S_i') - \alpha(S_j')] = (\mathbf{A_C}(\mathbf{b}_i - \mathbf{b}_j))\mathbf{z}.$$

Here we get

$$
\begin{aligned}
(\mathbf{A}_C(\mathbf{b}_1-\mathbf{b}_3))\mathbf{z} &= -1-\frac{2}{3}X+Y+\frac{1}{2}XY+\frac{1}{6}XY^3 \\
&= (1-Y)-X\left(\frac{2}{3}-\frac{1}{2}Y-\frac{1}{9}Y^3\right) < (1-Y) < 0 \\
(\mathbf{A}_C(\mathbf{b}_1-\mathbf{b}_5))\mathbf{z} &= -1-\frac{2}{3}X+Y+\frac{1}{2}XY+\frac{1}{9}XY^3+\frac{1}{18}XY^3Z^6 \\
&< (1-Y)-X\left(\frac{2}{3}-\frac{1}{2}Y-\frac{1}{9}Y^3\right) < (1-Y)-\frac{1}{18}X < 0 \\
(\mathbf{A}_C(\mathbf{b}_1-\mathbf{b}_7))\mathbf{z} &= -1+X < 0 \\
(\mathbf{A}_C(\mathbf{b}_1-\mathbf{b}_9))\mathbf{z} &= -3+\frac{4}{3}X+Y+\frac{1}{2}XY+\frac{1}{6}XY^3 < -3+\frac{4}{3}+1+\frac{1}{2}+\frac{1}{6} = 0 \\
(\mathbf{A}_C(\mathbf{b}_1-\mathbf{b}_{11}))\mathbf{z} &= -3+\frac{4}{3}X+Y+\frac{1}{2}XY+\frac{1}{9}XY^3+\frac{1}{18}XY^3Z^6 \\
&< -3+\frac{4}{3}+1+\frac{1}{2}+\frac{1}{9}+\frac{1}{18} = 0 \\
(\mathbf{A}_C(\mathbf{b}_1-\mathbf{b}_{13}))\mathbf{z} &= -2+Y+\frac{2}{3}XY+\frac{1}{3}XY^3 < -2+1+\frac{2}{3}+\frac{1}{3} = 0 \\
(\mathbf{A}_C(\mathbf{b}_1-\mathbf{b}_{15}))\mathbf{z} &= -3+X+Y+\frac{2}{3}XY+\frac{1}{3}XY^3 < -3+1+1+\frac{2}{3}+\frac{1}{3} = 0 \\
(\mathbf{A}_C(\mathbf{b}_1-\mathbf{b}_{17}))\mathbf{z} &= -2+X+\frac{1}{2}XY+\frac{4}{9}XY^3+\frac{1}{18}XY^3Z^6 \ < -2+1+\frac{1}{2}+\frac{4}{9}+\frac{1}{18} = 0 \\
(\mathbf{A}_C(\mathbf{b}_1-\mathbf{b}_{19}))\mathbf{z} &= -2+Y+\frac{2}{3}XY+\frac{2}{9}XY^3+\frac{1}{9}XY^3Z^6 < -2+1+\frac{2}{3}+\frac{2}{9}+\frac{1}{9} = 0 \\
(\mathbf{A}_C(\mathbf{b}_1-\mathbf{b}_{21}))\mathbf{z} &= -3+X+Y+\frac{2}{3}XY+\frac{2}{9}XY^3+\frac{1}{9}XY^3Z^6 \\
&< -3+1+1+\frac{2}{3}+\frac{2}{9}+\frac{1}{9} = 0 \\
(\mathbf{A}_C(\mathbf{b}_1-\mathbf{b}_{23}))\mathbf{z} &= -2+X+\frac{1}{2}XY+\frac{7}{18}XY^3+\frac{1}{9}XY^3Z^6 < -2+1+\frac{1}{2}+\frac{7}{18}+\frac{2}{18} = 0 \\
(\mathbf{A}_C(\mathbf{b}_1-\mathbf{b}_{25}))\mathbf{z} &= -1+X < 0 \\
(\mathbf{A}_C(\mathbf{b}_1-\mathbf{b}_{27}))\mathbf{z} &= -3+\frac{4}{3}X+Y+\frac{1}{2}XY+\frac{1}{6}XY^3Z^6 < -3+\frac{4}{3}+1+\frac{1}{2}+\frac{1}{6} = 0 \\
(\mathbf{A}_C(\mathbf{b}_1-\mathbf{b}_{29}))\mathbf{z} &= -3+\frac{4}{3}X+Y+\frac{1}{2}XY+\frac{1}{6}XY^3Z^6 < -3+\frac{4}{3}+1+\frac{1}{2}+\frac{1}{9}+\frac{1}{18} = 0 \\
(\mathbf{A}_C(\mathbf{b}_1-\mathbf{b}_{31}))\mathbf{z} &= -2+Y+\frac{2}{3}XY+\frac{1}{3}XY^3 < -2+1+\frac{2}{3}+\frac{1}{3} = 0 \\
(\mathbf{A}_C(\mathbf{b}_1-\mathbf{b}_{33}))\mathbf{z} &= -3+X+Y+\frac{2}{3}XY+\frac{1}{3}XY^3Z^6 < -3+1+1+\frac{2}{3}+\frac{1}{3} = 0 \\
(\mathbf{A}_C(\mathbf{b}_1-\mathbf{b}_{35}))\mathbf{z} &= -2+X+\frac{1}{2}XY+\frac{4}{9}XY^3+\frac{1}{18}XY^3Z^6 \ < -2+1+\frac{1}{2}+\frac{4}{9}+\frac{1}{18} = 0 \\
(\mathbf{A}_C(\mathbf{b}_1-\mathbf{b}_{37}))\mathbf{z} &= -2+Y+\frac{2}{3}XY+\frac{2}{9}XY^3+\frac{1}{9}XY^3Z^6 < -2+1+\frac{2}{3}+\frac{2}{9}+\frac{1}{9} = 0 \\
(\mathbf{A}_C(\mathbf{b}_1-\mathbf{b}_{39}))\mathbf{z} &= -3+X+Y+\frac{2}{3}XY+\frac{2}{9}XY^3+\frac{1}{9}XY^3Z^6 < -3+1+1+\frac{2}{3}+\frac{2}{9}+\frac{1}{9} = 0 \\
(\mathbf{A}_C(\mathbf{b}_1-\mathbf{b}_{41}))\mathbf{z} &= -2+X+\frac{1}{2}XY+\frac{7}{18}XY^3+\frac{1}{9}XY^3Z^6 < -2+1+\frac{1}{2}+\frac{7}{18}+\frac{2}{18} = 0 \\
(\mathbf{A}_C(\mathbf{b}_1-\mathbf{b}_{43}))\mathbf{z} &= -3+X+\frac{4}{3}Y+\frac{1}{3}XY+\frac{1}{3}XYZ^6 < -3+X+\frac{4}{3}+\frac{1}{3}+\frac{1}{3} = 0 \\
(\mathbf{A}_C(\mathbf{b}_1-\mathbf{b}_{45}))\mathbf{z} &= -3+X+\frac{4}{3}Y+\frac{1}{3}XY+\frac{1}{3}XYZ^6 < -3+X+\frac{4}{3}+\frac{1}{3}+\frac{1}{3} = 0 \\
(\mathbf{A}_C(\mathbf{b}_1-\mathbf{b}_{47}))\mathbf{z} &= -1+Y-\frac{1}{2}XY+\frac{4}{9}XY^3+\frac{1}{18}XY^3Z^6 \\
&= -1+Y-XY\left(\frac{1}{2}-\frac{4}{9}Y^2-\frac{1}{18}Y^2Z^6\right) < -1+Y < 0
\end{aligned}
$$

$$
\begin{aligned}
(\mathbf{A}_C(\mathbf{b}_1 - \mathbf{b}_{49}))\mathbf{z} &= -3 + X + \frac{4}{3}Y + \frac{1}{3}XY + \frac{2}{9}XY^3 + \frac{1}{9}XYZ^6 \\
&< -3 + 1 + \frac{4}{3} + \frac{1}{3} + \frac{2}{9} + \frac{1}{9} = 0 \\
(\mathbf{A}_C(\mathbf{b}_1 - \mathbf{b}_{51}))\mathbf{z} &= -3 + X + \frac{4}{3}Y + \frac{1}{3}XY + \frac{2}{9}XY^3 + \frac{1}{9}XYZ^6 \\
&< -3 + 1 + \frac{4}{3} + \frac{1}{3} + \frac{2}{9} + \frac{1}{9} = 0 \\
(\mathbf{A}_C(\mathbf{b}_1 - \mathbf{b}_{53}))\mathbf{z} &= -1 + Y - \frac{1}{2}XY + \frac{4}{9}XY^3 + \frac{1}{18}XY^3Z^6 \\
&= -1 + Y - X\left(\frac{1}{2}Y - \frac{4}{9}Y^2 - \frac{1}{18}Y^2Z^6\right) < -1 + Y < 0 \\
(\mathbf{A}_C(\mathbf{b}_1 - \mathbf{b}_{55}))\mathbf{z} &= -1 + X - \frac{2}{3}Y + \frac{5}{9}XY^3 + \frac{1}{9}XY^3Z^6 \\
&= -1 + X - Y\left(\frac{2}{3} - \frac{5}{9}Y^2 - \frac{1}{9}Y^2Z^6\right) < -1 + X < 0 \\
(\mathbf{A}_C(\mathbf{b}_1 - \mathbf{b}_{57}))\mathbf{z} &= -1 + X - \frac{2}{3}Y + \frac{5}{9}XY^3 + \frac{1}{9}XY^3Z^6 \\
&= -1 + X - Y\left(\frac{2}{3} - \frac{5}{9}Y^2 - \frac{1}{9}Y^2Z^6\right) < -1 + X < 0 \\
(\mathbf{A}_C(\mathbf{b}_1 - \mathbf{b}_{59}))\mathbf{z} &= -\frac{2}{3}XY + \frac{5}{9}XY^3 + \frac{1}{9}XY^3Z^6 = -XY\left(\frac{2}{3} + \frac{5}{9}Y^2 + \frac{1}{9}Y^2Z^6\right) < 0
\end{aligned}
$$

For the pseudocaterpillar, T_{67} is S_P.

$$
\begin{aligned}
(\mathbf{A}_P(\mathbf{b}_{67}-\mathbf{b}_{61}))\mathbf{z}_P &= -1+Y-\frac{1}{18}XY+\frac{1}{18}XYZ^6 \\
&= -1+Y-\frac{1}{18}XY(1-Z^6) < -1+Y < 0 \\
(\mathbf{A}_P(\mathbf{b}_{67}-\mathbf{b}_{62}))\mathbf{z}_P &= -2+X+\frac{2}{3}Y+\frac{5}{18}XY+\frac{1}{18}XYZ^6 < -2+1+\frac{2}{3}+\frac{5}{18}+\frac{1}{18}=0 \\
(\mathbf{A}_P(\mathbf{b}_{67}-\mathbf{b}_{63}))\mathbf{z}_P &= -2+X+\frac{2}{3}Y+\frac{5}{18}XY+\frac{1}{18}XYZ^6 < -2+1+\frac{2}{3}+\frac{5}{18}+\frac{1}{18}=0 \\
(\mathbf{A}_P(\mathbf{b}_{67}-\mathbf{b}_{64}))\mathbf{z}_P &= -1+Y-\frac{1}{18}XY+\frac{1}{18}XYZ^6 \\
&= -1+Y-\frac{1}{18}XY(1-Z^6) < -1+Y < 0 \\
(\mathbf{A}_P(\mathbf{b}_{67}-\mathbf{b}_{65}))\mathbf{z}_P &= -2+X+\frac{2}{3}Y+\frac{5}{18}XY+\frac{1}{18}XYZ^6 < -2+1+\frac{2}{3}+\frac{5}{18}+\frac{1}{18}=0 \\
(\mathbf{A}_P(\mathbf{b}_{67}-\mathbf{b}_{66}))\mathbf{z}_P &= -2+X+\frac{2}{3}Y+\frac{5}{18}XY+\frac{1}{18}XYZ^6 < -2+1+\frac{2}{3}+\frac{5}{18}+\frac{1}{18}=0 \\
(\mathbf{A}_P(\mathbf{b}_{67}-\mathbf{b}_{68}))\mathbf{z}_P &= -2+\frac{2}{3}X+\frac{2}{3}Y+\frac{2}{3}XY < -2+\frac{2}{3}+\frac{2}{3}+\frac{2}{3}=0 \\
(\mathbf{A}_P(\mathbf{b}_{67}-\mathbf{b}_{69}))\mathbf{z}_P &= -2+\frac{2}{3}X+\frac{2}{3}Y+\frac{2}{3}XY < -2+\frac{2}{3}+\frac{2}{3}+\frac{2}{3}=0 \\
(\mathbf{A}_P(\mathbf{b}_{67}-\mathbf{b}_{70}))\mathbf{z}_P &= -1+X-\frac{1}{18}XY+\frac{1}{18}XYZ^6 \\
&= -1+X-\frac{1}{18}XY(1-Z^6) < -1+X < 0 \\
(\mathbf{A}_P(\mathbf{b}_{67}-\mathbf{b}_{71}))\mathbf{z}_P &= -2+\frac{2}{3}X+Y+\frac{5}{18}XY+\frac{1}{18}XYZ^6 < -2+\frac{2}{3}+1+\frac{5}{18}+\frac{1}{18}=0 \\
(\mathbf{A}_P(\mathbf{b}_{67}-\mathbf{b}_{72}))\mathbf{z}_P &= -2+\frac{2}{3}X+Y+\frac{5}{18}XY+\frac{1}{18}XYZ^6 < -2+\frac{2}{3}+1+\frac{5}{18}+\frac{1}{18}=0 \\
(\mathbf{A}_P(\mathbf{b}_{67}-\mathbf{b}_{73}))\mathbf{z}_P &= -1+X-\frac{1}{18}XY+\frac{1}{18}XYZ^6 \\
&= -1+X-\frac{1}{18}XY(1-Z^6) < -1+X < 0 \\
(\mathbf{A}_P(\mathbf{b}_{67}-\mathbf{b}_{74}))\mathbf{z}_P &= -2+\frac{2}{3}X+Y+\frac{5}{18}XY+\frac{1}{18}XYZ^6 < -2+\frac{2}{3}+1+\frac{5}{18}+\frac{1}{18}=0 \\
(\mathbf{A}_P(\mathbf{b}_{67}-\mathbf{b}_{75}))\mathbf{z}_P &= -2+\frac{2}{3}X+Y+\frac{5}{18}XY+\frac{1}{18}XYZ^6 < -2+\frac{2}{3}+1+\frac{5}{18}+\frac{1}{18}=0
\end{aligned}
$$

For the balanced tree, T_{76} is S_B. An interesting case is comparing T_{76} and T_{103}. The difference will tend to be large for Y and Z near 1 and X near 0. This corresponds to y and z near 0 and large x.

$$
\begin{aligned}
&(\mathbf{A}_P(\mathbf{b}_{76}-\mathbf{b}_{103}))\mathbf{z}_P > 0 \\
&\iff -1-\frac{2}{3}X+\frac{5}{3}YZ-\frac{1}{6}XYZ+\frac{1}{6}XY^3Z > 0 \\
&\iff X\left(-\frac{2}{3}-\frac{1}{6}YZ+\frac{1}{6}Y^3Z\right) > 1-\frac{5}{3}YZ
\end{aligned}
$$
